# Unraveling the dynamics of emotional regulation and parental warmth across early childhood: prediction of later behavioral problems

**DOI:** 10.1038/s41598-025-06846-5

**Published:** 2025-07-02

**Authors:** Aimé Isdahl-Troye, Paula Villar, María Álvarez-Voces, Estrella Romero

**Affiliations:** 1 Department of Psychology, University of Cyprus, Nicosia, Cyprus; 2 Institute of Psychology (IPsiUS), Universidade de Santiago de Compostela, A Coruña, Spain

**Keywords:** Behavioral problems, Child emotion regulation, Parental warmth, RI-CLPM, Early childhood, Psychology, Risk factors

## Abstract

The impact of behavioral problems on children’s psychosocial health underscores the importance of identifying early predictors. Research in developmental psychopathology highlights emotion regulation (ER) and parenting as critical processes for fostering childhood adjustment, though their transactional dynamics remain underexplored. This study examines the bidirectional relationship between child ER and parental warmth during early childhood and investigates how their interconnected development predicts behavioral problems by school age. The sample, drawn from the longitudinal ELISA project, comprised 2,341 children (48.2% girls; ages 3–10). A Random Intercept Cross-Lagged Panel Model (RI-CLPM) was employed to distinguish individual-level differences from within-person changes, providing a more nuanced perspective. Results indicated stable individual differences in ER and parental warmth over time, even accounting for sociodemographic and temperamental factors. At the within-person level, bidirectional relations emerged: children’s emotional competencies influenced parental warmth, while parental warmth fostered children’s emotional skills. These reciprocal cycles predicted later behavioral outcomes. Specifically, carry-over changes in ER significantly predicted both conduct and emotional problems, whereas carry-over changes in parental warmth predicted only emotional problems. Findings are discussed in terms of the relevance of the interplay between ER and warmth in preventing internalizing and externalizing behaviors during childhood.

**Supplementary Information:**

The online version contains supplementary material available at 10.1038/s41598-025-06846-5.

## Introduction

Behavioral problems that emerge in early childhood often persist into later developmental stages, disrupting children’s adaptive functioning and overall well-being^1,2^. Research in developmental psychopathology seeks to identify early indicators of behavioral difficulties and to clarify the pathways and mechanisms underlying their progression, with the key aim of reducing both their frequency and their negative impact on children’s mental health^3^.

Externalizing behaviors are among the most prominent and distinctive early-onset difficulties in childhood^4^. Conduct problems, in particular, are a leading cause of referrals to child mental health services^5^, given their high prevalence and the substantial disruptions within family dynamics and broader social environments. Early childhood behavioral problems may also manifest as internalizing behaviors^6^. Although emotional difficulties tend to be less visible than conduct problems, they can also interfere with adaptive functioning, impacting academic performance and the development of peer relationships^7^. Addressing early behavioral problems is critical to preventing a potential cascade of adverse psychosocial outcomes^8^. This pressing need is heightened by the fact that child and adolescent mental health was already a significant public health challenge before COVID-19^9^, and has been further exacerbated by the crisis, with younger children being disproportionately affected^10^. In response, recent research has increasingly focused on identifying predictors of developmental difficulties in early childhood^11,12^. Specifically, transdiagnostic research has demonstrated that co-occurring conduct and emotional problems can emerge as early as the preschool years^13–15^, reflecting shared underlying factors and processes associated with externalizing and internalizing behaviors^16–18^. Among these shared factors, the interaction between child emotion regulation and parenting quality has garnered substantial research interest.

### Emotion regulation and parenting as predictors of behavioral problems

Developmental psychopathology research highlights emotion regulation (ER) as pivotal to children’s adaptive development, as it reflects their ability to manage stressful demands and emotional challenges in a socially appropriate, adaptive, and flexible manner^19–21^. According to the comprehensive view of Eisenberg et al.^22^, ER is a process involving both internal and external mechanisms. While much research has primarily examined internal processes, with temperamental self-regulation as a central aspect^23^, Eisenberg et al.^22^ also stressed the critical role of external influences, particularly positive parenting, in fostering emotional competence during early childhood^24,25^.

Similar to ER, parenting quality is a well-established predictor of children’s behavioral outcomes^26,27^. Traditionally, research on externalizing problems often highlighted the role of negative parenting practices, such as coercion and inconsistency^28,29^. Recently, attention has shifted toward examining the role of positive parenting in promoting children’s emotional and social competencies^30^, particularly ER^31,32^, which in turn, is linked to fewer behavioral problems in early childhood^33–35^.

Recent findings supporting the relationship between parenting and ER in promoting childhood adjustment^36–38^ are in line with contemporary transactional perspectives that emphasize the reciprocal nature of parent-child interactions, where both parents and children influence each other throughout development^39,40^. Heuristic models by Eisenberg et al.^41^ and Morris et al.^25^ provide frameworks for understanding the bidirectional roles of parents and children in ER socialization and adaptative behavior. On one hand, these transactional models highlight how parental emotional support, particularly in early childhood, fosters children’s ER by creating a family environment characterized by strong emotional bonds and high-quality parent-child interactions. On the other hand, they acknowledge that children actively engage in their emotional socialization, shaping both their emotional development and outcomes^42–44^. Thus, these perspectives emphasize the interdependent nature of parent-child dynamics, wherein “parent effect” and “child effect” are viewed as interwoven processes^45,46^.

Although studies exploring the bidirectional parent-child interactions have increased in recent years^31^, relatively few have focused on early childhood. Notable exceptions include studies by Spinrad et al.^47^, Tiberio et al.^48^ and Neppl et al.^49^, which examine the reciprocal dynamics between parenting and child-driven effects from the preschool years. These studies are particularly relevant to the present investigation as they examine the longitudinal associations between parenting and children’s ER—specifically effortful control (EC)—using path modelling to capture reciprocal influences while accounting for the stability of each construct over time. Regarding the main findings of these studies, Spinrad et al.^47^ found that maternal warmth predicted toddlers’ EC one year later, but effects faded over time. Similar findings from Tiberio et al.^48^ linked early EC development primarily to concurrent parenting and prior EC stability. However, evidence of transactional effects emerged only in late childhood, highlighting EC’s stability and concurrent parenting as dominant factors. Both studies emphasized the need for further research on bidirectional dynamics during critical developmental transitions. Drawing a distinction, in their study, Neppl et al.^49^ introduce an innovative extension of the traditional Cross-Lagged Panel Model (CLPM), the Random Intercept Cross-Lagged Panel Model (RI-CLPM^50^), to address unobserved heterogeneity, specifically individual differences, that is not measured in classic CLPM. Their analysis of 220 rural families with children aged 3 to 5 found that positive parenting influenced children’s EC, but no reciprocal effects were observed. Additionally, they did not find evidence that within-person changes in parenting or EC predicted externalizing problems. The authors acknowledge that their results contrast with studies supporting a mediational process linking parenting, self-regulation, and behavioral problems^51,52^, urging further research with diverse samples and broader measures to substantiate the transactional hypothesis. For instance, He et al.^53^ examined two samples of Chinese children (average age 9.6) with and without oppositional defiant disorder (ODD). Their findings indicated a unidirectional child effect in the non-ODD group, consistent with the “child effect”, and a transactional dynamic in the ODD group. Overall, their results are congruent with prior research^19,47,48^, emphasizing the significance of child characteristics in later childhood and the importance of parental influence in cases of maladjustment.

### Exploring parent-child influences through a multilevel perspective

Adopting a multilevel approach to examining parent-child dynamics provides a nuanced understanding of the transaction between individual and contextual factors. CLPM are commonly used in developmental research due to their ability to model causal relationships and handle longitudinal data. However, CLPMs cannot determine whether changes in a predictor precede changes in an outcome. The RI-CLPM addresses this limitation by distinguishing between-person and within-person effects, thereby helping to clarify the directionality of causal relationships^50^. Specifically, RI-CLPM controls for trait-like individual differences allowing researchers to examine whether deviations from an individual’s baseline in one variable predict subsequent changes in another variable^54^.

Given these advantages, several longitudinal studies have adopted RI-CLPM to explore the bidirectional parent-child interactions^55,56^. However, its application in investigating transactional relationships between parenting and emotion regulation in childhood remains limited. Notably, the studies by Neppl et al.^49^ and He et al.^53^ are pioneering in applying this approach to examine the associations between parental warmth and child ER. However, differences across these studies - including variations in cultural contexts that frame parent-child interactions, developmental stages assessed (i.e., early vs. late childhood), and discrepancies in the measures used to evaluate positive parenting (i.e., observational vs. self-reported) and child emotion regulation (i.e., effortful control vs. emotional regulation competence) - highlight the need for further research. Adopting a multilevel perspective in future studies will be crucial in clarifying which findings hold developmental significance.

## The current study

The present study utilizes four waves of longitudinal data from the ELISA project (Estudio Longitudinal para una Infancia Saludable; see https://www.personalitydevelopmentcollaborative.org/project-page-elisa/ for details) to explore the relationship between child ER and positive parenting, and their combined influence on subsequent developmental outcomes during early childhood. Advancing beyond previous research, we leverage the RI-CLPM to overcome the constraints of traditional cross-lagged models^53^.

The primary objective of the study is to test the potential bidirectional relationship between child ER and parental warmth. Specifically, we hypothesized: (1) At the between-person level, trait-like differences in child ER and parental warmth will be positively related; (2) At the within-person level, higher-than-expected levels of ER will be associated with higher-than-expected levels of parental warmth within a time point and vice versa; in other words, the time-specific association between children´s ER and parental warmth will be positive; (3) At the within-person level, the longitudinal relation between parental warmth and children’s ER will be reciprocal, such that higher-than-expected levels of parental warmth at a given time point will be associated with lower-than-expected levels of ER in the future and vice versa; in other words, spill-over effects from parental warmth to child ER and spill-over effects from child ER to parental warmth will be positive.

An additional objective is to examine the extent to which the co-development of child ER and parental warmth during early childhood predicts behavioral problems by the time children reach school age. We hypothesize that this reciprocal relationship process could serve as a mechanism not only supporting competent ER development, but also promoting adaptive behavioral development, as evidenced by a lower incidence of both externalizing and internalizing problems.

By exploring these dynamic interactions, the study aims to offer valuable insights into how emotion regulation and positive parenting practices may jointly contribute to the development of emotional and conduct outcomes, with implications for early intervention and support.

## Results

### Descriptive statistics

First, we analyzed differences in the study variables across assessment points. Table 1 displays the means, standard deviations, scale range and the paired t-test for the repeated measures. In general, results indicated that child ER means steadily increased over the assessment period, whereas the means for parental warmth showed a decrease over time. However, the effect sizes of these differences were generally small, with Cohen’s *d* values ranging from 0.10 to 0.26 (in absolute terms), suggesting overall limited change across time points.

### Longitudinal relations between child ER and parental warmth

Table 2 presents the Pearson correlation matrix for all variables studied. Overall, the patterns of association indicate stability in the core constructs, with significant positive correlations observed between child ER and parental warmth across the three waves (*P* < 0.001).

The Intra-Class Correlation (ICC) for child ER and parental warmth was calculated to determine whether sufficient within-person variability existed across waves, a crucial step before conducting the RI-CLPM. The ICC for child ER was 0.61, indicating that 61% of the variance in ER over time is due to between-person differences. Similarly, the ICC for parental warmth was 0.60, suggesting that 60% of the variance is attributable to between-person differences, with the remaining variance reflecting within-person fluctuations. Therefore, performing the RI-CLPM was justified, as 39–40% of the variance for these variables could be attributed to within-person (state-like) differences across waves.

**Table 1:** Descriptive statistics of study variables in all assessment points and rate differences across assessment points (i.e., T1-T2, T2-T3).

	T1		T2		T1-T2	T3		T2-T3	T4	
	Mean (SD)	Range (Min, Max)	Mean (SD)	Range (Min, Max)	t (df)	Mean (SD)	Range (Min, Max)	t (df)	Mean (SD)	Range (Min, Max)
ER	1.70 (0.64)	0.00, 4.00	1.75 (0.67)	0.00, 4.00	-4.26***(1897)	1.91 (0.70)	0.00, 4.00	-10.65*** (1679)	—	—
WARM	4.70 (0.38)	1.00, 4.00	4.67 (0.38)	2.83, 5.00	4.28*** (1872)	4.62 (0.42)	2.67, 5.00	4.74*** (1653)	—	—
CP	—	—	—	—	—	—	—	—	0.26 (0.29)	0.00, 1.60
ES	—	—	—	—	—	—	—	—	0.47 (0.42)	0.00, 2.00
EMO	3.03 (0.82)	1.00, 5.00	—	—	—	—	—	—	—	—

ER = Emotion Regulation; WARM = Parental Warmth; CP = Conduct Problems; ES = Emotional Symptoms; EMO = Emotionality. All values were rounded to two decimals. *P < 0.05, **P < 0.01, ***P < 0.001.

**Table 2:** Pearson’s correlation matrix all study variables.

	1	2	3	4	5	6	7	8	9	10	11	12
**1.** REG (T1)	1											
**2.** REG (T2)	**0.63** ^***^	1										
**3.** REG (T3)	**0.58** ^***^	**0.61** ^***^	1									
**4.** WARM (T1)	**0.14** ^***^	**0.11** ^***^	**0.12** ^***^	1								
**5.** WARM (T2)	**0.14** ^***^	**0.16** ^***^	**0.18** ^***^	**0.58** ^***^	1							
**6.** WARM (T3)	**0.12** ^***^	**0.13** ^***^	**0.16** ^***^	**0.54** ^***^	**0.60** ^***^	1						
**7.** CP (T4)	**-0.36** ^***^	**-0.45** ^***^	**-0.47** ^***^	-0.06	**-0.12** ^***^	**-0.09** ^**^	1					
**8.** ES (T4)	**-0.13** ^***^	**-0.17** ^***^	**-0.20** ^***^	-0.02	-0.03	**-0.09** ^**^	**0.27** ^***^	1				
**9.** GEN	**-0.05** ^*^	**-0.07** ^**^	**-0.09** ^***^	0.01	0.03	0.02	**0.10** ^**^	-0.04	1			
**10.** SES	**0.08** ^***^	**0.10** ^***^	0.04	-0.01	-0.03	0.02	**-0.11** ^***^	**-0.11** ^***^	0.01	1		
**11.** AGE	0.05*	0.00	0.02	-0.06**	-0.07**	-0.07**	-0.00	0.02	-0.02	-0.05*	1	
**12.** EMO	-0.46***	-0.34***	-0.33***	-0.03	-0.04	-0.07**	0.30***	0.24***	-0.04	-0.12***	0.02	1

ER = Emotion Regulation Skills; WARM = Parental Warmth; ES = Emotional Symptoms; CP = Conduct Problems; GEN = Child Gender (1 = girls, 2 = boys, i.e., boys compared to girls); AGE = Child Age Group (1 = youngest, 2 = oldest, i.e., oldest compared to youngest); SES = Family SES; EMO = Emotionality. All values were rounded to two decimals. *P < 0.05, **P < 0.01, ***P < 0.001.

**Fig. 1:**
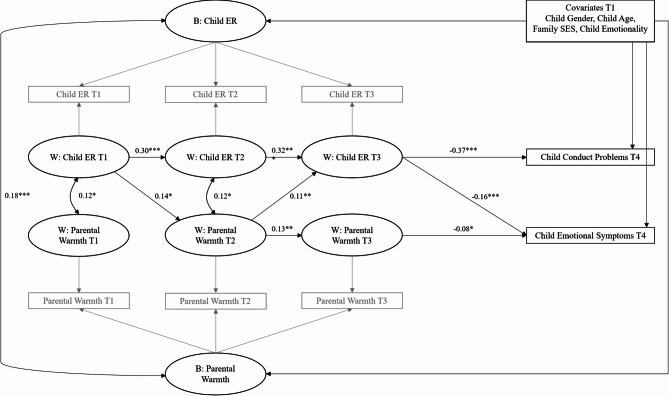
Relationships between Child ER and Parental Warmth using RI-CLPM, with the within-child variations in ER and Warmth at T3 predicting Child Conduct Problems and Emotional Symptoms at T4. Standardized parameters. B = Between-Person, W = Within-Person. Child Gender, Age, Emotionality and Family SES at baseline were added as control variables on the effects over Child Conduct Problems/Emotional Symptoms and over the between-child differences in Child ER and Parental Warmth (i.e., Random Intercepts). All values were rounded to two decimals. *P < 0.05, **P < 0.01, ***P < 0.001.

Consequently, the RI-CLPM was applied to examine the relationship between child ER and parental warmth, including the influence of within-person fluctuations in these variables on problem behaviors. The model controlled for the covariates: child age, gender, family SES, and emotionality. The model fit indices met the standard criteria for an adequate fit: *χ²* (35) = 22.025, *P* = 0.04; CFI = 0.954; TLI = 0.922; RMSEA = 0.048; SRMR = 0.041. The between-person correlation between the random intercept factors of child ER and parental warmth was small (unstandardized b < 0.30) but statistically significant (*P* < 0.001), suggesting that children with higher levels of emotional regulation were typically in families where caregivers also displayed higher warmth. This relationship remained significant even after controlling for child age, gender, family SES, and emotionality. Moreover, covariates explained some of the between-person variability in child ER and parental warmth. Specifically, boys had lower rates of ER compared to girls, the oldest children had higher rates of ER compared with youngest but had less parental warmth, and children with higher emotionality tended to show less ER and have less parental warmth compared to children with lower emotionality. For detailed unstandardized estimates, standard errors, and covariate effects, refer to Supplementary Table S1 online. As illustrated in Fig. 1, the results show autoregressive and cross-lagged effects after adjusting for between-person differences. Specifically, the autoregressive paths for child ER were significant, indicating that children who were relatively high (or low) on ER at a given assessment at one time point were likely to maintain similar levels at the next point. In parallel, parental warmth demonstrated positive carry-over effects from T2 to T3, indicating that caregivers who exhibited relatively high (or low) levels of warmth during the second wave tended to maintain those warmth levels at the third wave, reflecting the stability of parenting behaviors over time.

In terms of cross-lagged effects, the results demonstrated that child ER and parental warmth contributed to each other: child ER at T1 predicted higher parental warmth at T2, and higherparental warmth at T2 predicted higher child ER at T3. These pathways indicate that within-child variability in ER at baseline was associated with within-family variability in parental warmth at T2, and that within-family variability in parental warmth at T2 was associated with future within-child variability in ER. Spill-over effect sizes from child ER to parental warmth, and from parental warmth to child ER, ranged from medium to large^57^.

### Predictive paths to behavioral problems

Finally, we examined the predictive effects of the co-development of child ER and parental warmth during early childhood on behavioral problems at school age (see Fig. 1). Our results indicated that both the path from child ER to conduct problems at T4 and the path from child ER to emotional symptoms at T4 were statistically significant (*β* = -0.37, SE = 0.03, *P* < 0.001; *β*= -0.16, SE = 0.04, *P* < 0.001). Specifically, when children exhibited higher levels of ER than their baseline at T1, they tended to maintain these higher levels of ER at subsequent waves (T2 and T3), which, in turn, was negatively associated with behavioral problems at T4. Furthermore, the path from parental warmth to emotional symptoms at T4 was also statistically significant (*β* = -0.08, SE = 0.03, *P* < 0.05). These pathways indicate that carry-over changes in child ER significantly predicted both externalizing and internalizing problems, while carry-over changes in parental warmth predicted only internalizing problems.

## Discussion

This study employed the RI-CLPM to investigate the bidirectional relationship between child ER and parental warmth in early childhood, disentangling between-person differences from within-person changes for a clearer understanding of their dynamics. Additionally, the model explored how preschool ER and parental warmth predict conduct and emotional problems at school age, addressing limitations of traditional cross-lagged models and advancing insights into the underlying developmental processes.

The initial focus of the study was to test the relationship between children’s ER and parental warmth at the between-person level. We first hypothesized that stable trait-like aspects of children´s ER and parental warmth would be positive and significantly related to each other, such that children with higher ER would tend to belong to families where the primary caregiver exhibits higher levels of parental warmth. Our results supported this hypothesis, which is aligned with previous evidence in this field^48,49,58^. Specifically, the model showed that there are stable individual differences in the ER of young children across time, and also in the warmth displayed by their parents. Additionally, results point to some predictors of the tendency to show consistently high or low ER. Thus, age seems to be an involved factor, in that older children tend to show higher ER than younger children, in accordance with previous studies on the normative changes of ER^59,60^. As for gender, results showed that girls display higher levels of ER, which also concurs with some previous studies on the self-regulation of girls and boys at early childhood^61^. On the other hand, child’s emotionality predicts stable tendencies in ER, reinforcing former claims about the role of this reactive temperamental trait in determining ER^62^. When the predictors of parental warmth are examined, results also highlight the role of age and emotionality: parents of younger children tend to show warmer interactions, and parents of more reactive children (i.e., higher in emotionality) tend to show less warmth; this result draws attention to the potential of early child’s temperament for conditioning of affective parent-child relationships^63^.

Therefore, the analyses identified between-persons variability in the disposition of children to show ER and of parents to show warmth in their interactions with children. The delineation of such tendencies, which are predicted by sociodemographic and temperamental factors, emphasizes the need to disaggregate between- and within- person effects when analyzing the relationship between warmth and ER. Families with warmer parents seem to have children with higher ER, but this is not enough for elucidating whether the change in one of the variables involves a change in the other. The focus needs to be placed on the individual variations with respect to their stable tendencies, and in our study, the RI-CLPM was a useful tool for this aim.

At the within-person level, our findings also supported our expectations regarding the positive association between children´s ER and parental warmth. Specifically, our results showed that changes in ER were associated with changes in warmth, i.e., when warmth increases in the parents, the ER of their children increases too. This result, drawn from a restrictive analysis like RI-CLPM, supports the assumption that the closeness and the affective interactions between parents and children might be crucial for understanding the development of self-regulation^31,36,47^, and more specifically, emotional regulation^32,34,37^. Consistent with our hypotheses, our results showed that warmth and ER are also related in the reverse direction: when a child exhibits a higher-than-expected level of ER, a corresponding increase in parental warmth is observed. Thus, it is not just the family atmosphere that may affect the emotional development of children; children’s characteristics and behaviors also appear to help shape the quality of parent-child relations, in line with models and studies that underscored the active role of children in the socialization process^39^.

Thus, our results show that the longitudinal relationship between parental warmth and children’s ER would be reciprocal. Intra-individual variability in ER could be influenced by the primary caregiver’s efforts to maintain warm relationships with the child. Our findings confirmed that parental warmth during early childhood stimulates the child´s ER observed one year later. This is consistent with existing literature and prominent models^25,41^ emphasizing the importance of positive parental communication and behavior in the development of children´s emotional competence; if parents provide a nurturing environment, with emotional responsiveness and warm interactions, there seems to be a good groundwork for fostering ER in their children. Contrary to the premise outlined in our initial hypothesized model, this “parent effect” was not evident at baseline when the children were three years old. Nonetheless, the results indicated that higher levels of child ER at baseline increase parental warmth one year later.

Overall, according to our results, it seems that, within the family system, both parents and children can stimulate positive exchanges in a mutually reinforcing process. The emotional competences by children could impact the affective implication by parents; in turn, parents’ warmth would promote children’s emotional skills. This finding underscores the active role of children as agents in their own developmental process, highlighting that the socialization of ER is not a unidirectional force. Instead, children’s influence on parental warmth portrays a dynamic interplay consistent with the transactional perspective on human development^40^. Furthermore, such cycles of child-to-parent and parent-to-child influences are expected to impact the conduct and emotional outcomes as children grow up^41^.

Precisely, modelling the prediction of behavioral problems based on the interwoven development of warmth and ER was the second objective of this study. While some studies have examined the longitudinal relationships between parenting, child ER and early childhood problems^48,64^, few have adopted a bidirectional approach^49,53^ and explored both conduct and emotional difficulties^17^.

Our findings evidenced the significance of ER in predicting both externalizing and internalizing problems, consistent with previous evidence on the capacity of ER for explaining a wide range of psychosocial challenges^16^. Such results bolster the relevance of ER for developmental psychopathology, and support the role of ER as a shared source of influence for emotional and conduct problems in childhood^14,15,18^.

Results also showed the impact of parental warmth on later children’s problems. In this case, the effects were more specific: parental warmth preschool predicted fewer emotional problems at school age, in line with previous research on its impact on internalizing difficulties^27^. While positive parenting has also been linked to the development of conduct problems in previous studies^26^, this association was not found in our study, stressing the need for further investigation in this field.

In general terms, the analysis of conduct and emotional outcomes brings support for the effects of warmth and ER on later problems. At a theoretical level, results on warmth agree with classical models on the role of emotional security for developing emotional well-being^65^, and on the role of emotional support for dealing with stress^66^ and fostering emotional resilience^67^. On the other hand, results on ER, which impacts both emotional and conduct problems, support transdiagnostic models, which underscore common mechanisms across multiple psychopathological problems^68^. Particularly, ER has been central to transdiagnostic models in adults^69^ and has also been proposed as a pivotal cross-cutting factor in children’s psychopathology^16,70^.

Summing up, building on the contributions of prior studies^33,35^, our findings on the reciprocal relationship between warmth and ER suggest that this transactional process not only facilitates the development of effective ER but also supports healthier behavioral patterns. Moreover, our results demonstrated that, besides consistent dispositions in terms of ER and warmth, there are also changes throughout the preschool years, and, remarkably, such variations can predict behavioral problems at school age. Thus, it is possible that a young child with the tendency to display low ER, increases their ER above what is expected, and this change could reduce the likelihood of developing behavioral difficulties at a later stage. Thus, our results provide new evidence to the expanding field of ER as a driving factor for different kinds of maladjustment in children.

In terms of practical implications, although more longitudinal research is needed on this topic, the fact that conduct and/or emotional problems might respond to previous changes in warmth or ER endorses the potential usefulness of interventions on family dynamics and children’s emotional competences for prevention of children’s problems both in the internalizing and externalizing spectra. For example, programs on emotional socialization parenting^71^, which engage parents and children in recognition, validation and regulation of emotional states, could hold promise as resources for promotion of mental health from the preschool years. The interplay between warmth and ER might also suggest the helpfulness of multi-component programs, which combine family modules for positive parenting and school modules for promotion of emotional competence in children; the efficacy of such programs for prevention of later psychological problems has been, in fact, evidenced by previous research^72,73^.

However, the findings of this study should be viewed in the light of some limitations. First, this study relied on parent-reported data, and, thus, the effect of shared-method variance cannot be dismissed; also, despite the efficiency of parent reports for assessment of both parents’ and young children’s behaviors^74^, potential caveats in parents’ reports, related with expectations, emotional states, or with the cross-situational variability of children’s behavior^75^ need to be considered. Second, in this study most of informants were mothers, partially reflecting the more prominent role of women in caregiving; nevertheless, the socializing behaviors of fathers, and the differential parenting patterns among mothers and fathers should be addressed in further research, following some previous studies in this field^49,76^. Third, like most longitudinal studies, ours could not avoid participants’ attrition over time; the presence of differential attrition based on SES advises caution when extrapolating the results across social settings. Also, further research should investigate on the generalizability across cultures and levels of risk (e.g., clinical or high-risk populations). Additionally, future research could strengthen the findings of this study by increasing the number of measurements and analyzing the transactional processes across different developmental periods. While the RI-CLPM was suitable for estimating time-specific, bidirectional within-person effects, it does not account for either general or individual trends in development^77^. In this study, the overall changes observed in child ER and parental warmth, although aligned with normative expectations, appeared to be small. Supplementary growth curve analyses (see Supplementary Material S2 online) reinforced these results and further indicated that interindividual variability in change was limited, supporting the appropriateness of the RI-CLPM for addressing the research questions in the present study. Nevertheless, future studies with more time points and wider developmental windows may benefit from models that integrate growth and transactional dynamics, such as the ALT-SR^78^. Beyond expanding the temporal span of assessments, incorporating additional variables (e.g., parents’ traits, parenting stress, family emotional expressiveness) would provide a deeper account of how children’s emotional regulation develops within the family.

Despite these limitations, the current results contribute to the existing literature on the transactional dynamics of parenting and self-regulation, with a particular focus on intraindividual variability over time in families of young children^49^. Our study reinforces the role of family socialization in this process, even accounting for sociodemographic and temperamental influences. Yet, the picture of an “influential child”^79^ is also sketched by our results, illustrating how ER may carve interactions and environments, and bolstering ER as a critical target for promotion of psychosocial health in children.

## Methods

### Sample

The present study used data from 2,341 preschool children (48.2% girls) from the general population who participate in the ELISA project. The ELISA project is an ongoing prospective study conducted in Galicia (North-western Spain) to analyze developmental pathways of child conduct, emotional behavior and psychosocial adjustment, starting from early childhood. Participating children were recruited from 72 schools located in 27 urban, suburban and rural areas in Galicia. Children ranged in age from 3 to 6 at T1 (M = 4.24, SD = 0.90), 4–7 at T2 (M = 5.34, SD = 0.92), 4–8 at T3 (M = 6.31, SD = 0.92) and 6–10 at T4 (M = 8.40, SD = 0.95). Most of the children were Spanish (approximately 98%).

Data were collected from questionnaires fulfilled by one caregiver per household at three each time point: baseline (T1 = 2016–2017) plus three subsequent assessment points (T2 = 2017–2018; T3 = 2018–2019; T4 = 2020–2021). At the first wave, 92.4% of fathers and 77.2% of mothers worked outside the home. In terms of the parents’ level of education, 14% of mothers and 30.1% of fathers had finished compulsory school education, 38.7% and 38.2% had completed a post-compulsory education, and 31.2% and 47.5% had concluded university studies.

As with any longitudinal study, sample attrition is unavoidable. In this study, attrition rates were 10.7% between T1 and T2, 19.1% between T1 and T3, and 48.4% between T1 and T4. Notably, the primary reason for the increased attrition at T4 was the impact of the COVID-19 pandemic, which affected data collection during the initial post-pandemic phase. Comparisons between children who participated in all waves and those who missed follow-ups revealed no statistically significant differences in age (*F*(5, 2362) = 0.54, *P* = 0.750), gender (*χ²*(5) = 6.03, *P* = 0.303), or initial levels of conduct (*F*(5, 2224) = 0.57, *P* = 0.720) and emotional problems (*F*(5, 2225) = 0.87, *P* = 0.500). However, significant differences emerged in socioeconomic status (SES) (*F*(5, 2233) = 17.29, *P* < 0.001), with children who participated in all waves showing higher SES levels, a trend consistent with prior longitudinal studies.

### Procedure

The ELISA project received approval from the Spanish Ministry of Science, Innovation and Universities and the Bioethics Committee of the University of Santiago de Compostela. All procedures adhered to the guidelines and regulations established by the approving institutions, ensuring compliance with the Declaration of Helsinki and relevant ethical codes. Informed written consent was obtained from each participating child’s primary caregiver or legal guardian before administering questionnaires. Data confidentiality was strictly maintained, and participation was entirely voluntary, with no incentives or compensation provided.

Initially, 126 schools in Galicia were contacted, including 77.6% public, 17.1% charter, and 5.2% private schools. Of these, 72 schools agreed to participate. Families of approximately 5,300 preschoolers were invited to join, with 25–50% of families from each school opting in. Primary caregivers, mostly mothers (87.3%), completed standardized questionnaires annually in the spring (March–June) at each data collection point. Teachers were responsible for distributing and collecting questionnaires under the supervision of project coordinators designated at each school by the ELISA lead team. We endeavored to standardize the administration of questionnaires to ensure consistency across the diverse schools involved in the study. This included harmonizing the order of scale presentation and maintaining uniformity in the timing and setting of administration. For further methodological details, see https://www.personalitydevelopmentcollaborative.org/project-page-elisa/.

### Measures

All measures were based on parent reports, and the questionnaires had been previously translated, adapted, and validated in studies conducted within the Spanish context.

### Child and parent indicators in the bidirectional model

#### Child emotional regulation

Child ER was assessed using the Emotion Regulation Skills subscale of the Social Competence Scale (SCS) – Parental Version (Conduct Problem Prevention Research Group)^80^. This subscale consists of six items related to children’s self-control skills (e.g., “Copes well with failure”, “Can calm down when excited”, “Controls temper when disagreement”). Responses are rated on a five-point Likert scale, from 0 (“not at all”) to 4 (“very well”).

The SCS has demonstrated strong psychometric properties in community and at-risk samples, spanning preschool to second grade children^80,81^. It also shows positive validity correlations with parent ratings on the Emotion Regulation Checklist (ERC^82^). Prior studies using this subscale to assess children’s ER have reported satisfactory reliability^64,83,84^. In the current study, the *Emotion Regulation Skills* subscale demonstrated good internal consistency, with Cronbach’s alpha coefficients of 0.80 (T1), 0.81 (T2), and 0.84 (T3).

#### Parental warmth

Parental warmth was measured using a shortened 6-item version of the original Warmth Scale from the Child Rearing Questionnaire^85^. This reduced version demonstrated adequate model fit in the Longitudinal Study of Australian Children^86^ for assessing the frequency of positive emotional tone in parent-child interactions (e.g., “You hug or hold the child for no particular reason”, “You have warm, close times together with this child”). Items were rated on a 5-point scale (1 = never to 5 = always). In the present study, Cronbach’s alpha coefficients ranged from 0.81 to 0.82 across assessment points, indicating good internal consistency.

### Child outcomes

#### Behavioral problems

Conduct and emotional problems were assessed using two subscales from the *Strengths and Difficulties Questionnaire* (SDQ) Spanish version^87^: the conduct problems scale (e.g., “Often has temper tantrums or hot tempers”, “Often fights with other children or bullies them”) and the emotional symptoms scale (e.g., “Many worries, often seems worried”, “Often unhappy, downhearted, or tearful*”*). Each scale consists of five items rated on a 3-point Likert scale to reflect how applicable the attribute is to the child’s behavior (0 = not true, 1 = somewhat true, 2 = certainly true). The SDQ has demonstrated robust reliability and validity for identifying emotional and behavioral problems in children and adolescents aged 3–16 years^88,89^, with strong evidence supporting its use in Spanish community samples of preschoolers^90^. In the current study, Cronbach’s alpha coefficients were 0.62 for the conduct problems scale and 0.72 for the emotional symptoms scale.

### Examination of covariables

#### Age and gender

In this study, we controlled for the effects of child age group (3–4 years old [younger] vs. 5–6 years old [older]), as self-regulatory skills typically become more sophisticated with age, reflecting normative improvements in ER competence during early childhood^59,60^. Similarly, we accounted for gender (boys vs. girls), given evidence of gender differences in emotion expression and regulation during childhood, as well as potential interaction effects between gender and age^61,91^.

#### Socioeconomic status

Family socioeconomic status (SES) was included as a covariate, consistent with prior research identifying SES as a risk factor for behavioral issues^92^. SES was indexed through parental education level and family economic status. Parents’ education level was averaged from the father’s and mother’s education on a six-point scale (1 = no basic studies to 6 = postgraduate studies). Family economic status was based on reported income (1 = serious financial difficulties to 4 = well off) and financial solvency (1 = never worried to 5 = daily concern). The composite SES measure, based on *Z*-transformed variables (*α* = 0.65), demonstrated good psychometric properties in prior studies^14,93^.

#### Emotionality

We controlled for child emotionality, a temperamental trait related to emotional reactivity, which is an important predictor of both child maladjustment^23,94^ and the quality of the parenting environment^63^. Emotionality was assessed using the Spanish version of the emotionality scale from Buss and Plomin’s Emotionality Activity Sociability (EAS) Temperament Survey for Children: Parental Ratings^95^. This scale, consisting of 5 items rated on a five-point Likert scale, from 1 (“never like this”) to 5 (“always like this”), evaluates children’s tendency to show distress or become upset easily (e.g., “often fusses and cries”, “get upset easily”). The alpha coefficient for this scale was 0.71.

### Statistical analysis

Descriptive statistics and Pearson’s correlations of the variables in question were calculated using IBM SPSS Statistics 27. Regarding descriptive statistics, the observed variables included in the cross-lagged model (i.e., child ER and parental warmth) were repeated measures, thus we examined whether the means of variables at adjacent time points significantly differed (i.e., increased or decreased) by using *t*-Tests for paired samples. For Pearson’s correlations, the strengths of the correlations were interpreted using Cohen’s standards^96^. Additionally, intraclass-correlation coefficients (ICC) for ER and parental warmth were also explored. The ICCs were calculated to quantify the within-person variance for child ER and parental warmth over time.

The Random Intercept Cross-Lagged Panel Model (RI-CLPM) in Mplus version 7.4^97^ was used to examine reciprocal effects between child ER and parental warmth over a three time-point assessment period^98^. The between-person component is accounted for by the inclusion of a random intercept (i.e., a latent variable whose factor loadings are constrained to 1). Thus, the autoregressive and cross-lagged parameters of this model are related exclusively to the within-person fluctuations in both the child’s ER and the parent’s warmth variables. In other words, the RI-CLPM allowed us to estimate individual deviations in the child and parent from the expected values of ER and warmth (respectively) – individual deviations which were not captured by the between-person latent variables (i.e., random intercepts).

RI-CLPM was calculated by including the child’s problem behaviors at T4 as distal outcomes (extensions) in the model and additional variables to control for the random intercepts and child outcomes (see Supplementary Figure S1 online). The potential control variables or covariates here were child age, gender, family SES, and emotionality. Of these, the ones that showed significant correlations with the model indicators and outcomes were included in the model. There are different possible ways to navigate the model specification of a RI-CLPM^99^. For the purposes of this study, we estimated the effects of the within-child variations in ER and the within-parent variations in warmth at T3 on problem behaviors (i.e., emotional symptoms and conduct problems) at T4.

The model was estimated using MLR for maximum likelihood estimation with robust standard errors. Missing data on covariates were handled by mentioning their variances in the MODEL command in Mplus. Model fit was evaluated using the Chi-square statistic (*χ²*), the Comparative Fit Index (CFI), the Tucker-Lewis Index (TLI), the Standardized Root Mean Square Residual (SRMR), and the Root Mean Squared Error of Approximation (RMSEA). CFI and TLI values greater than or equal to 0.95 and SRMR and RMSEA values less than or equal to 0.05 were considered appropriate, whereas CFI and TLI values greater than 0.90, and RMSEA and SRMR less than 0.08 were considered acceptable^100,101^. Together with the information provided by the statistical analysis, the interpretability and theoretical relevance of the model were also considered.

## Electronic supplementary material

Below is the link to the electronic supplementary material.

Supplementary Material 1

## Data Availability

The datasets generated and analysed during the current study are available from the corresponding author on reasonable request.
