# Supplementary material for: Unraveling the dynamics of emotional regulation and parental warmth across early childhood: prediction of later behavioral problems
Source: Sci Rep. 2025 Jul 2;15:23294. doi: 10.1038/s41598-025-06846-5 (PMC12222833; doi:10.1038/s41598-025-06846-5)
Supplement: Supplementary file 1 — Supplementary Material 1 [file 41598_2025_6846_MOESM1_ESM.pdf]

Supplementary material: Unraveling the dynamics of emotional regulation and parental warmth across early childhood: Prediction of later behavioral problems

Parameters	Est.	(SE)	Std. Est.	(SE)
**Within-Person Part**				
Lagged effects				
ER T2 → T2	**0.36*** **	**(0.07)**	**0.30*** **	**(0.06)**
ER T2 → T3	**0.33*** **	**(0.05)**	**0.32*** **	**(0.05)**
WARM T1 → T2	-0.03	(0.08)	-0.03	(0.08)
WARM T2 → T3	**0.16***	**(0.07)**	**0.13***	**(0.06)**
Cross-Lagged effects				
ER T1 → WARM T2	**0.08***	**(0.04)**	**0.14***	**(0.06)**
ER T2 → WARM T3	0.02	(0.03)	0.03	(0.05)
WARM T1 → ER T2	0.01	(0.09)	0.01	(0.05)
WARM T2 → ER T3	**0.25** **	**(0.08)**	**0.11** **	**(0.04)**
Distal Outcomes (Extensions)				
ER T3 → CP (T4)	**-0.20*** **	**(0.02)**	**-0.37*** **	**(0.03)**
ER T3 → ES (T4)	**-0.13*** **	**(0.03)**	**-0.16*** **	**(0.04)**
WARM T3 → CP (T4)	-0.02	(0.04)	-0.02	(0.04)
WARM T3 → ES (T4)	**-0.12***	**(0.05)**	**-0.08***	**(0.03)**
Correlations				
T1	0.01	(0.01)	**0.12***	**(0.06)**
T2	**0.02***	**(0.01)**	**0.12***	**(0.05)**
T3	0.01	(0.01)	0.07	(0.03)
**Between-Person Part**				
ER & WARM	**0.02** **	**(0.01)**	**0.18** **	**(0.05)**
Covariates				
GENDER → ER	**-0.11*** **	**(0.02)**	**-0.12*** **	**(0.02)**
SES → ER	0.03	(0.02)	0.04	(0.02)
AGE → ER	**0.05***	**(0.02)**	**0.06***	**(0.02)**
AGE → WARM	**-0.05** **	**(0.02)**	**-0.08** **	**(0.02)**
EMO (T1) → ER	**-0.33*** **	**(0.01)**	**-0.59*** **	**(0.03)**
EMO (T1) → WARM	**-0.02***	**(0.01)**	**-0.06***	**(0.02)**
**Controlling Distal Outcomes**				
GENDER → CP (T4)	**0.06*** **	**(0.02)**	**0.11*** **	**(0.02)**
SES → CP (T4)	**-0.03***	**(0.01)**	**-0.08** **	**(0.03)**
SES → ES (T4)	**-0.05** **	**(0.02)**	**-0.09** **	**(0.03)**
EMO (T1) → CP (T4)	**0.11*** **	**(0.01)**	**0.32*** **	**(0.02)**
EMO (T1) → ES (T4)	**0.13*** **	**(0.02)**	**0.24*** **	**(0.03)**

Table S1. Unstandardized and standardized estimates, and standard errors, of the RI-CLPM. ER = Emotion Regulation; WARM = Parental Warmth; ES = Emotional Symptoms; CP = Conduct Problems; GENDER = Child Gender (1 = girls, 2 = boys, i.e., boys compared to girls); AGE = Child Age Group (1 = youngest, 2 = oldest, i.e., oldest compared to youngest); SES = Family SES; EMO = Emotionality. All values are rounded to two decimals. *$P < 0.05$, **$P < 0.01$, ***$P < 0.001$.

	Slope Mean (Estimate / *P*)	Slope Variance (Estimate / *P*)	RMSEA	CFI
**WARM**	-0.037 / < 0.001	0.004 / 0.305	0.244	0.259
**ER**	0.098 / < 0.001	0.013 / 0.151	0.199	0.511

Table S2. Summary of latent growth curve models (LGCMs) estimated for parental warmth and emotion regulation across three time points.

ER = Emotion Regulation. WARM = Parental Warmth. Estimates refer to the latent slope mean and slope variance for each construct. *P*-values correspond to model-estimated parameters. RMSEA and CFI reflect model fit indices.

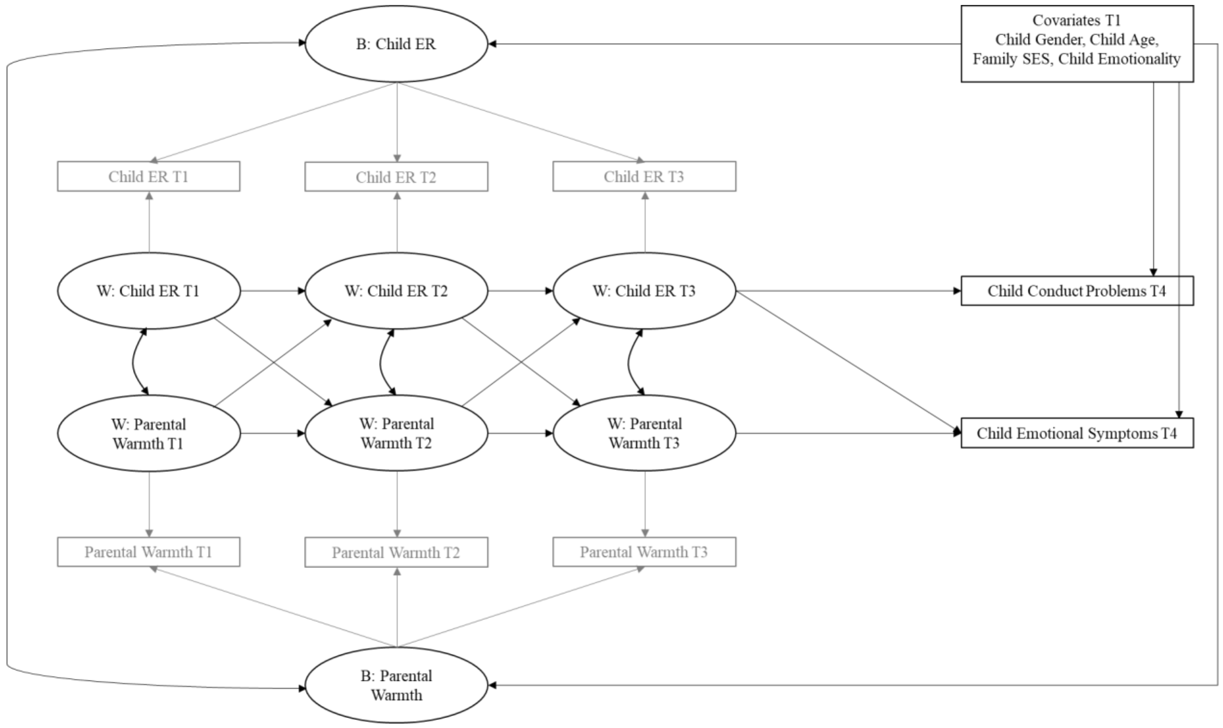

Figure S1. Model diagram of the relationship between Child ER and Parental Warmth using RI-CLPM, with the within-child variations in ER and Warmth at T3 predicting Child Conduct Problems and Emotional Symptoms at T4.

B = Between-person component, W = Within-person component. Child Gender, Age, Emotionality and Family SES at baseline were added as control variables on the effects over Child Conduct Problems/Emotional Symptoms and over the between-child differences in Child ER and Parental Warmth (i.e., random intercepts).
